# Rapid video-based deep learning of cognate versus non-cognate T cell-dendritic cell interactions

**DOI:** 10.1038/s41598-021-04286-5

**Published:** 2022-01-11

**Authors:** Priya N. Anandakumaran, Abigail G. Ayers, Pawel Muranski, Remi J. Creusot, Samuel K. Sia

**Affiliations:** 1 Department of Biomedical Engineering, Columbia University, New York, NY 10027 USA; 2 Department of Medicine, Division of Hematology/Oncology, Columbia University Irving Medical Center, New York, NY 10032 USA; 3 Columbia Center for Translational Immunology, Columbia University Irving Medical Center, New York, NY 10032 USA; 4 Department of Medicine and Naomi Berrie Diabetes Center, Columbia University Irving Medical Center, New York, NY 10032 USA

**Keywords:** High-throughput screening, T cells, Biomedical engineering, Cellular imaging

## Abstract

Identification of cognate interactions between antigen-specific T cells and dendritic cells (DCs) is essential to understanding immunity and tolerance, and for developing therapies for cancer and autoimmune diseases. Conventional techniques for selecting antigen-specific T cells are time-consuming and limited to pre-defined antigenic peptide sequences. Here, we demonstrate the ability to use deep learning to rapidly classify videos of antigen-specific CD8^+^ T cells. The trained model distinguishes distinct interaction dynamics (in motility and morphology) between cognate and non-cognate T cells and DCs over 20 to 80 min. The model classified high affinity antigen-specific CD8^+^ T cells from OT-I mice with an area under the curve (AUC) of 0.91, and generalized well to other types of high and low affinity CD8^+^ T cells. The classification accuracy achieved by the model was consistently higher than simple image analysis techniques, and conventional metrics used to differentiate between cognate and non-cognate T cells, such as speed. Also, we demonstrated that experimental addition of anti-CD40 antibodies improved model prediction. Overall, this method demonstrates the potential of video-based deep learning to rapidly classify cognate T cell-DC interactions, which may also be potentially integrated into high-throughput methods for selecting antigen-specific T cells in the future.

## Introduction

Interactions between cognate T cells and antigen-presenting dendritic cells (DCs) are a critical component of the cell-mediated adaptive immune response and peripheral tolerance^1,2^. For understanding disease progression, the absence of T cell priming by cognate DCs or other antigen-presenting cells (APCs) prevents immune-mediated control of tumors^3^, while the inappropriate priming of self-reactive T cells by cognate DCs is involved in the progression of autoimmune diseases such as type I diabetes^2^. Furthermore, the identification of antigen-specific T cells is also necessary for the development of adoptive cell therapies such as T cell receptor (TCR)-engineered T cell therapies, which involve the delivery of T cells that are reactive against particular antigens, such as tumor antigens in the context of cancer^4^. Hence, a number of bulk^5,6^ and microfluidic^7–10^ methods have been developed in order to identify antigen-specific T cells from the vast T cell repertoire. However, many of these require hours of co-culture between T cells and DCs (or other APCs) prior to classification, and require known peptide sequences (Supplementary Table S1). Some approaches use calcium imaging to measure T cell activation, but calcium imaging also has some limitations, as calcium responses in T cells can be low or absent for low-affinity cognate interactions^7^ and increases in calcium may be unrelated to the binding of antigens^8^.

Recently, machine learning-based approaches have been used for T cell classification, in which quantitative variables such as autofluorescence lifetime^11^, or morphological variables^12^ are extracted from images and used to classify quiescent and activated T cells, or cognate and non-cognate T cell-DC contacts, respectively. However, these models are both limited by the need for long T cell activation times, while the latter omits critical temporal information that can be used to improve classification accuracy (Supplementary Table S1).

Here, we use deep learning to classify cognate, antigen-specific T cells by leveraging the unique interaction dynamics between cognate and non-cognate T cells-DCs observed as naive T cells scan DCs for the presence of the cognate pMHC complex in lymphoid organs^13^. Contact between T cells and cognate DCs results in changes in T cell morphology and motility, whereby T cells either immediately, or after some period of transient interactions called kinapses, flatten against the cognate DC and form stable, long-lasting interactions called synapses^14,15^. T cells interacting with non-cognate DCs, on the other hand, scan and make transient interactions with many DCs, and do not exhibit the same changes as those making stable cognate interactions^13^. We hypothesize that a deep learning model, aided by anti-CD40 antibodies (aCD40), can classify CD8^+^ T cells interacting with cognate DCs, and CD8^+^ T cells interacting with non-cognate DCs, based on their distinct interaction dynamics in terms of morphology and motility.

## Results

### Overview of video processing procedure and deep learning pipeline

**Figure 1:**
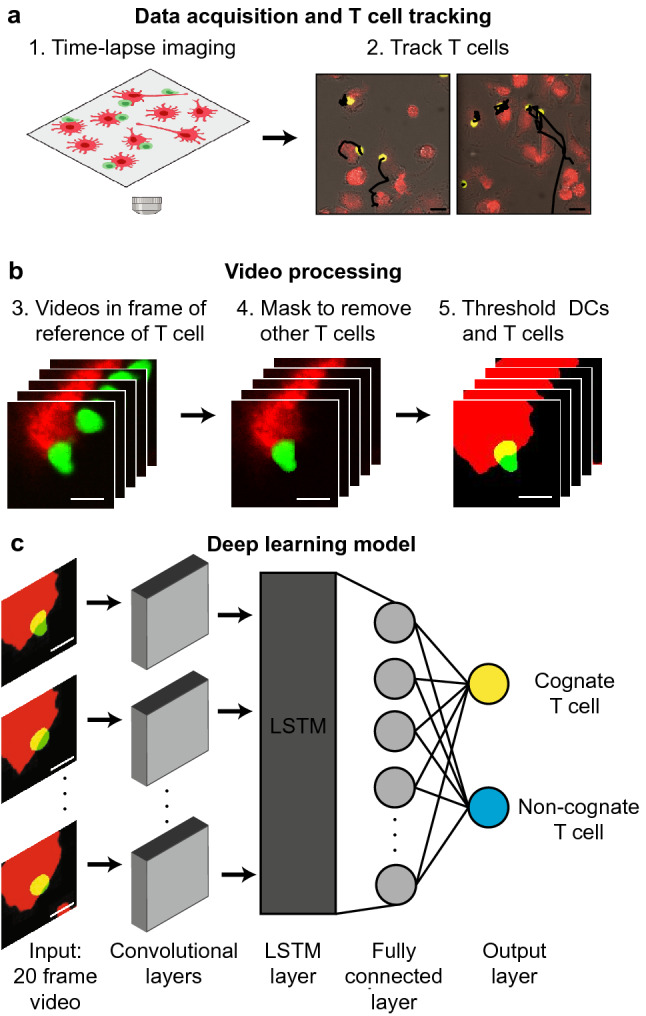
Schematic diagram of video processing and deep learning-based binary classifier. (**a**) Schematic of time-lapse microscopy of interacting T cells (green) and DCs (red) (created with Biorender.com) and subsequent automated T cell tracking (tracks in black). Scale bar is 20 µm. (**b**) Process to generate individual, thresholded, 101 × 101 pixel videos in the frame of reference of each T cell. Scale bar is 10 µm. (**c**) Overview of the deep learning-based binary classifier. The model consists of a feature extractor (with time distributed CNN, batch normalization and max pooling layers), LSTM model, fully connected layer, and final softmax classification layer to classify T cells as making cognate or non-cognate interactions with DCs. Scale bar is 10 µm.

We studied T cell-DC interactions using OT-I TCR transgenic mice, an established model system^16^. In this TCR transgenic mouse model, CD8^+^ T cells recognize a specific peptide sequence from ovalbumin, OVA_257-264_ (N4) peptide (SIINFEKL), restricted to H-2K^b^ MHC class I. OT-I CD8^+^ T cells were co-cultured with N4 peptide-presenting DCs (N4-DCs) or un-pulsed DCs, and the cells were imaged every 1–2.5 min, for up to 1.5 h (Table 1 for experimental details). We used an automated cell tracking software (Fig. 1a) to track each T cell, then cropped a moving 101 × 101 pixel region around the centroid of the T cell, and concatenated the images to form a video in the frame of reference of the T cell. We then applied a mask to remove any neighboring T cells present in the frame to ensure that only the centered T cell of interest was visible. The T cell and DC channels were then thresholded to remove any imaging-related or experiment-specific noise and artifacts (Fig. 1b). Finally, to ensure that each video in the data set had the same number of frames, while maximizing information, each video was reduced to a total of 20 frames which were uniformly sampled from the original video. By varying this sampling rate and the experimentally captured frame rate, we ensured that the model could robustly classify T cells, independently of the sampling rate.

**Table 1:** Experimental details.

Experiment	Time between frames (min)	Max number of frames	+ aCD40		− aCD40	
			Antigen-specific	Non-antigen specific	Antigen-specific	Non-antigen specific
1. OT-I	1.5	45	197 (N4)	256	195 (N4)	196
2. OT-I	2	30	–	–	227 (N4)	314
3. OT-I	2	40	179 (N4)	195	190 (N4)	174
4. OT-I	2	40	205 (Q4)	–	–	–
5. OT-II	1.5	41	219	153	178	178
6. NY8.3	1	61	94	253	109	205
7. NY8.3	1.5	46	295	304	224	227

A subset of the videos of cognate and non-cognate interactions were then used to train a binary classifier. The binary classifier is a convolutional neural network (CNN)-long short-term memory (CNN-LSTM) network, consisting of CNN layers for feature extraction, an LSTM model to maintain memory of each frame, a fully connected layer, and a final classification layer (Fig. 1c). The output of the model is the probability that the T cell in the inputted video is making cognate interactions with DCs (in which case it is considered an antigen-specific T cells), or non-cognate interactions with DCs.

### Use of aCD40 to improve discrimination of cognate vs. non-cognate interactions

**Figure 2:**
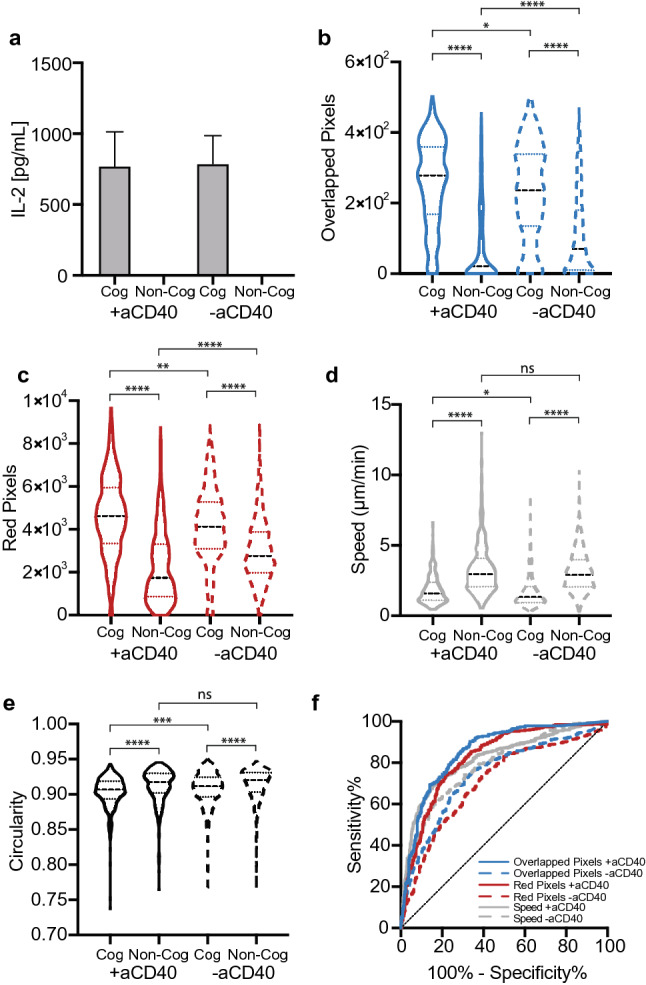
Using traditional image analysis methods to classify cognate vs. non-cognate T cell-DC interactions, with or without aCD40. (**a**) IL-2 secretion by cognate and non-cognate T cells incubated with and without aCD40 (mean ± standard deviation). (**b**)–(**e**) Distributions of (**b**) overlapped pixels (**c**) red pixels (**d**) speed and (**e**) circularity for cognate and non-cognate OT-I T cells incubated with or without aCD40, averaged over all of the frames in the video. (**f**) ROC curve quantifying the ability of the different metrics to discriminate between cognate and non-cognate T cells.

We hypothesized that the incubation of DCs with aCD40, prior to the addition of T cells, could amplify the differences in interaction dynamics between cognate and non-cognate T cell-DCs. One possible mechanism is that CD40 agonists enable DC licensing, leading to increased expression of co-stimulatory and adhesion molecules^17^ and secretion of inflammatory cytokines^18^, which may reinforce cognate interactions. To assess the effect of aCD40 on cognate T cell-DC interactions, and T cell activation, we measured IL-2 secretion on day 3 of T cell-DC co-culture, and found similar levels of IL-2 secretion between co-cultures of cognate T cell-DCs incubated with or without aCD40 (Fig. 2a).

### Traditional image analysis methods for classification of OT-I T cell-DC interactions

We first applied traditional image analysis techniques for classifying T cell-DC interactions. Specifically, we first quantified the total area of the T cells and DCs in each frame by determining the total number of binarized green and red pixels, respectively (Supplementary Fig. S1). The average number of overlapped pixels, between T cells and DCs, that are present in both the red and green channels (Supplementary Fig. S1) was then quantified as an indicative measurement of proximity between the two cell types (Fig. 2b). The average number of red pixels was also quantified because cognate T cells spend more time in close proximity to DCs than non-cognate T cells (Fig. 2c). Note that the average number of red pixels in the pre-processed/un-cropped videos is the same in all of the conditions (Supplementary Fig. S2), and so any differences in red pixels between cognate and non-cognate T cells in Fig. 2c is not due to differences in initial DC numbers, but rather due to the greater propensity of cognate T cells to be in contact with DCs. Speed (Fig. 2d) is a common classification metric because cognate T cells typically have lower speeds due to their longer interaction times with DCs, while non-cognate T cells rapidly scan DCs with minimal long-term interactions^19^. As such, we also quantified the percentage of time each T cell interacts with a DC, as an indicative measurement of interaction time (Supplementary Fig. S3). Finally, we also used average circularity as a classification metric in order to account for the differences in morphology between cognate and non-cognate T cells as they interact with DCs (Fig. 2e).

As expected based on what is known about the motility and morphology of cognate T cells in comparison to non-cognate T cells as they interact with DCs, there were significantly more overlapped pixels and red pixels, and significantly lower speed and circularity in cognate T cells in comparison to non-cognate T cells, both with and without anti-CD40 (Fig. 2b-e). The overlapped pixel data was also used to visualize T cell interactions with DCs over time, and it can be seen that cognate T cells spend more time in contact with DCs in comparison to non-cognate T cells (Supplementary Fig. S3). The degree of discrimination between cognate and non-cognate T cells was quantified by the area under the curve (AUC) of a receiver-operator curve (ROC) (Table 2, Fig. 2f) which plots the true positive rate (sensitivity) vs. false positive rate (1-specificity) for a variety of cut-off values. While circularity provided a mediocre discrimination, the rest of the metrics were able to more robustly distinguish between cognate and non-cognate T cells, especially in the presence of aCD40 (summarized in Table 2). We found that the overlapped pixels metric, with aCD40, provided the best discrimination between cognate and non-cognate T cells, with an AUC of 0.86. This was comparable to the machine learning model developed by Liarski et al.^12^, who trained a model to classify static images of T cells interacting with cognate or non-cognate DCs using extracted distance and morphology parameters, and achieved an AUC of 0.84.

**Table 2:** Summary of OT-I T cell AUCs.

	AUC	
	+ aCD40	− aCD40
Red pixels	0.83	0.71
Overlapped pixels	0.86	0.75
% of time each T cell interacts with DCs	0.85	0.76
Speed	0.79	0.82
Circularity	0.64	0.59
Deep Learning	0.91	0.75

With the exception of speed, the addition of aCD40 resulted in a higher AUC, and thus, greater discrimination between cognate and non-cognate T cells for all of the metrics tested, in comparison to the absence of aCD40 (Table 2). The addition of aCD40 served to improve the classification by enhancing the differences between cognate and non-cognate T cells. For example, the addition of aCD40 significantly decreased the number of overlapped pixels and red pixels in non-cognate T cells, while it significantly increased the same metrics in cognate T cells (Fig. 2b, c). The aCD40-mediated increase in red and overlapped pixels in cognate cultures may be due to the reinforcement of cognate interactions from DC licensing. Although it is possible that aCD40 could also block interactions between CD40 and CD40 ligand in non-cognate interactions (similar to how the deletion of ICAM-1 signaling in DCs reduces long-term binding with non-cognate T cells^20^), CD40 was likely newly synthesized in the T cells as the initial incubation period with the antibody was short (10 min); future studies will be required to establish the exact mechanism of aCD40.

Furthermore, while the addition of aCD40 to non-cognate T cell-DCs did not significantly impact T cell speed or circularity (shape), it significantly decreased the circularity of cognate T cells. Unexpectedly, we found that the addition of aCD40 significantly increased the speed of cognate T cells, which could be attributed to changes in DC speed, since the DCs were also motile, and thus T cell speed could not be easily decoupled from DC speed. However, in the majority of cases, the addition of aCD40 further amplified the differences between cognate and non-cognate T cell-DCs, which improved the classification accuracy.

### Deep learning for classification of OT-I T cell-DC interactions

**Figure 3:**
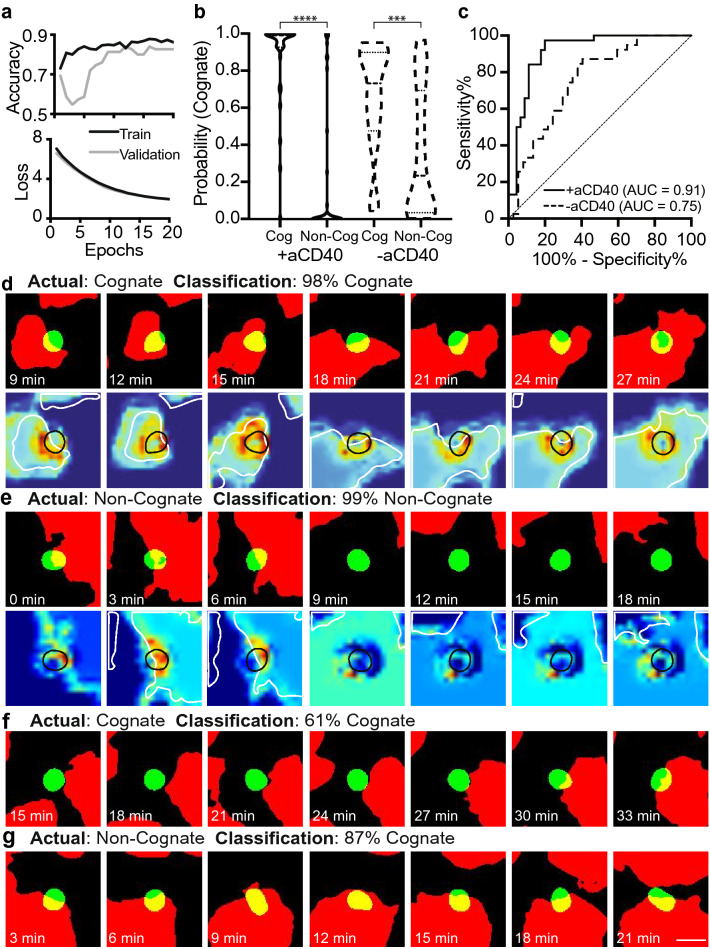
Using deep learning to classify cognate vs. non-cognate T cell-DC interactions, with or without aCD40. (**a**) Accuracy and loss curves during training and validation of the deep learning model using high affinity cognate and non-cognate OT-I T cells incubated with aCD40. (**b**) Predicted probability that an OT-I T cell is cognate using models trained and tested on datasets with or without aCD40. (**c**) ROC curves quantifying the ability of the deep learning models to discriminate between cognate and non-cognate T cells based on the probabilities predicted in (**b**). (**d**)–(**e**) Examples of T cells which were correctly, and with a high probability classified as (**d**) cognate, and (**e**) non-cognate. (**f**)–(**g**) Examples of T cells which were (**f**) correctly classified as cognate with a lower probability and (**g**) incorrectly classified as cognate. In (**d**) and (**e**), the Grad-CAM heatmaps visualize the regions important for the designated classification in red (DC outline is overlaid in white, and T cell outline is overlaid in black). Scale bar is 10 µm.

After establishing a baseline degree of classification using image analysis techniques, we trained the CNN-LSTM deep learning model (Fig. 1c) to more accurately classify videos of cognate or non-cognate OT-I T cells, either with or without aCD40 (referred to as + aCD40 or -aCD40, respectively). Robust training without overfitting was achieved by performing data augmentation, optimizing the architecture of the model, and including regularization terms. Two separate binary classifiers were trained to distinguish cognate and non-cognate T cells using samples with or without aCD40. In each case, 80% of the videos were used for training, 10% for validation, and 10% for testing, with the + aCD40 classifier achieving training and validation accuracies around 85%, with steadily decreasing loss (Fig. 3a). Overall, the + aCD40 binary classifier provided better discrimination between cognate and non-cognate T cells (Fig. 3b), yielding an AUC of 0.91, while the -aCD40 model yielded an AUC of 0.75 (Fig. 3c). In line with the non-deep learning methods, the presence of aCD40 resulted in more effective classification, and as such, in the work below, we only consider the models trained and tested using samples with aCD40.

In order to interpret the + aCD40 model, we examined videos from the test set which were correctly predicted as being cognate (Fig. 3d) and non-cognate (Fig. 3e), with a high probability. Consistent with what is known about the interaction dynamics between cognate T cells and DCs, the correctly predicted cognate T cell remained in contact with the same DC for almost the entire duration of the video (Fig. 3d), while the non-cognate T cell quickly detached from a DC after 3 frames of contact (Fig. 3e). We also implemented Gradient-weighted Class Activation Mapping (Grad-CAMs) to visualize the regions activated by the penultimate convolution block which are important to the designated classification in red (bottom of Fig. 3d, e, with DCs overlaid in white, and the T cell overlaid in black)^21^. According to the Grad-CAMs, the areas of importance for cognate T cell classification was the overlapped region between T cells and DCs, which was consistent with our previous, simple analysis (Fig. 3d bottom). Whereas, when identifying non-cognate T cells, the regions of importance were less consistent (Fig. 3e bottom).

To further probe the results of the model, we also examined examples of T cells which were correctly predicted as cognate but with a lower confidence (Fig. 3f), and those which were incorrectly predicted as cognate but with high confidence (Fig. 3g). The reduced predicted probability (0.61) of being cognate in Fig. 3f may have been due to the lack of substantial overlap between the T cell and DC, despite being next to each other for at least 5 frames. Whereas the non-cognate T cell in Fig. 3g was likely incorrectly predicted as being cognate, and with a high probability (0.87) due to the substantial overlap between the T cell and DC. The Grad-CAMs and analysis of inaccurately or incorrectly predicted videos demonstrate the importance of overlap between T cells and DCs for accurate classification. However, given that the binary classifier had a higher AUC (0.91) than classifying based on overlapped pixels alone (AUC = 0.86), the deep learning model likely incorporates other information, other than simply the number of overlapped pixels.

### Performance of model, without adjustment of weights, to classify other types of T cell-DC interactions

**Figure 4:**
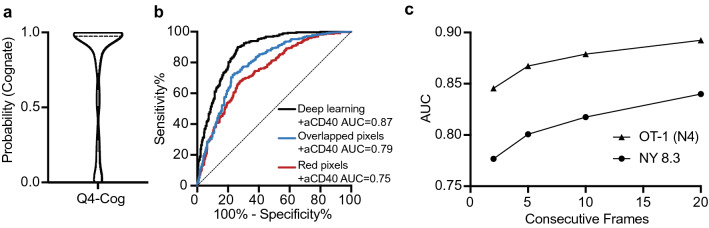
+aCD40 deep learning model trained on one set of interacting T cells and DCs accurately classifies other sets of interacting T cells and DCs. (**a**) Testing of the + aCD40 deep learning model (previously trained on videos of OT-I T cells interacting with un-pulsed, or high affinity N4 peptide-pulsed DCs (N4-DCs)) on T cells interacting with DCs presenting the lower affinity Q4 peptide. (**b**) ROC curves quantifying the ability of the +aCD40 deep learning model, overlapped pixels and red pixels to classify cognate or non-cognate NY8.3 T cells (cultured with aCD40). (**c**) Assessment of the ability of the deep learning model to classify high affinity cognate- and non-cognate OT-I and NY8.3 T cells when trained and tested on videos of varying length.

We explored the performance of this method, specifically using the previously trained + aCD40 deep learning model without any adjustments in weights, for classifying other types of cognate and non-cognate T cell-DCs interactions. First, we tested the model using videos of OT-I T cells interacting with DCs pulsed with the Q4 peptide (Q4-DCs), which have a lower affinity to OT-I CD8^+^ T cells in comparison to N4-DCs. It has been previously demonstrated that T cells interacting with Q4-DCs remained motile albeit slower, while T cells interacting with N4-DCs were arrested^19^. Consistent with previous observations, we found that T cells interacting with Q4-DCs had a mean speed in between that of T cells interacting with N4-DCs, and T cells interacting with non-cognate DCs (Supplementary Fig. S4). Despite these differences in T cell speed, the interaction dynamics of T cells interacting with Q4-DCs were nearly indistinguishable to T cells interacting with N4-DCs in terms of overlapped pixels and red pixels (Supplementary Fig. S4). By testing the binary classifier with cognate T cells interacting with Q4-DCs, we found that the model was able to accurately classify the lower affinity cognate T cell-DC interactions as well (Fig. 4a).

Next, we assessed the ability of the model to be generalized to CD8^+^ T cells from a different mouse strain. In particular, we tested the same model using CD8^+^ T cells from NY8.3 TCR transgenic mice, which are reactive against an epitope from the pancreatic islet cell antigen islet-specific glucose-6-phosphatase catalytic subunit related protein (IGRP) in the context of H-K^d^ MHC Class I, and are involved in the development of Type 1 diabetes in NOD mice. The aCD40 + model was able to discriminate well between cognate and non-cognate NY8.3 CD8^+^ T cells, yielding an AUC of 0.87 (Fig. 4b). Furthermore, the deep learning model was able to differentiate between cognate and non-cognate NY8.3 T cells better than the average number of overlapped pixels (AUC = 0.79) or the average number of red pixels alone (AUC = 0.75) (Fig. 4b).

Nevertheless, we observed some limitations of this method. In particular, this model (even with + aCD40) was not able to classify CD4^+^ T cells (in contrast to previously tested CD8^+^ T cells) from OT-II TCR transgenic mice (Supplementary Fig. S5). Speed, overlapped pixels and red pixels similarly resulted in mediocre discrimination between cognate and non-cognate OT-II CD4^+^ T cells (Supplementary Fig. S5). This lack of generalizability to CD4^+^ T cells is likely due to differences in the interaction dynamics between CD8^+^ T cells and DCs (via peptide-MHCI complexes) and CD4^+^ T cells and DCs (via peptide-MHCII complexes)^22^. Thus, a separate binary classifier likely has to be trained using cognate and non-cognate CD4^+^ T cells, in order to classify CD4^+^ T cells.

Finally, we assessed whether the deep learning model could be trained to classify shorter videos of OT-I high affinity cognate and non-cognate CD8^+^ T cells, and whether these models trained on shorter videos could still be generalized to the NY8.3 CD8^+^ T cells. Until this point, the deep learning model was trained and tested using 20-frame videos which were generated by evenly sampling the original videos, which corresponded to a time frame ranging from 20–80 min (Table 1). We next instead trained and tested new models with videos with shorter time frames. In particular, we generated new videos of interacting OT-I T cells and DCs consisting of 20, 10, 5 or 2 consecutive frames, where 20 consecutive frames corresponded to a time frame ranging from 20–40 min. As the number of consecutive frames in the videos was decreased, the AUC decreased as well, when tested on either the OT-I or NY8.3 T cells (Fig. 4c). Taken altogether, this demonstrates that temporal data is necessary, and that the classification accuracy increases with more frames, and with more time. However, the models which were trained and tested using 20 consecutive frames had an AUC (0.89 for OT-I and 0.84 for NY8.3), which was comparable to the models trained and tested using 20 evenly sampled frames (0.91 for OT-I and 0.87 for NY8.3). Thus, the AUC does eventually plateau, and an accurate classification could be made after up to 40 min of interactions, rather than up to 80 min.

## Discussion

This work demonstrated the ability to accurately classify videos of cognate and non-cognate CD8^+^ T cells based on their interactions with DCs in a rapid manner. We classified T cells within approximately 20–80 min of T cell-DC interactions, which is substantially faster than most classification methods (Supplementary Table 1), and is essential for high-throughput applications. Although further reducing the co-culture time would be beneficial, the imaging frequency would likely have to be increased, as the results showed that reducing the number of frames analyzed over a fixed time frame resulted in a decreased classification accuracy, while analyzing a fixed number of frames collected over different time frames resulted in comparable classification accuracies.

Although in this study we pulsed DCs with a known peptide sequence, this approach can potentially be done without this step if the isolated DCs (or APCs) are already presenting antigens (for example, tumor-associated antigens with unknown peptide sequences), unlike multimer- or artificial APC- based approaches, which require the peptide sequence to be known (Supplementary Table 1). This characteristic could be beneficial when the specific peptide sequence is unknown, such as for neoantigens, where sequencing methods are used to identify somatic mutations, but the specific peptide sequence can only be predicted using computational approaches^23^. This characteristic could also be useful if the DCs are pulsed with full antigens, or tumor cell lysates rather than peptides^24^.

The study demonstrated experimental and computational improvements in methodologies that resulted in significantly improved prediction. Experimentally, the addition of aCD40 amplified the differences between cognate and non-cognate interactions and significantly improved the classification accuracy. Although future work is required to better understand the mechanism through which aCD40 amplified the differences between cognate and non-cognate interactions, this addition of antibodies or small molecules to strengthen certain cellular behaviors, such as increasing specific interactions, or decreasing non-specific behavior, could be applied in other cell classification problems. Computationally, we found that thresholding the red (DC) and green (T cell) channels resulted in a more generalizable model. We previously trained models using videos of non-thresholded videos, as well as videos which also included the brightfield channel, however, those models quickly overfit to the training data, and were not able to be generalized to unseen data from different experiments (data not shown). By binarizing the red and green channels and removing the brightfield channel, we removed any imaging-related or experiment-specific noise and artifacts, and the model was able to generalize well to new data.

Future work can further develop the use of this method. Although the method identifies high- and low-affinity T cell-DC interactions, future work could be performed to validate the model for non-transgenic T cells, which may exhibit lower affinity for the target. This method can be used to identify cognate interactions between T cells and DCs to develop a deeper understanding of their role in disease progression, immunity, and tolerance^25^. Towards translational use, the classifier could be integrated into a microfluidic cell sorter^26^ to select, or enrich for rare antigen-specific T cells for TCR-engineered T cell therapies, which require the personalized, and high-throughput selection of antigen-reactive T cells.

## Methods

### Mice studies

All animal procedures were approved by the Columbia University Institutional Animal Care and Use Committee (IACUC) and all experiments were performed in accordance with relevant guidelines/regulations. C57BL/6 J (B6), C57BL/6-Tg(TcraTcrb)1100Mjb/J (OT-I), B6.Cg-Tg(TcraTcrb)425Cbn/J (OT-II), NOD/ShiLtJ (NOD), and NOD.Cg-Tg(TcraTcrbNY8.3)1Pesa/DvsJ (NY8.3) mice were purchased from The Jackson Laboratory (Bar Harbor, Maine).

### Cell culture

Bone marrow cells were harvested from the tibia and femur of mice, and they were pre-enriched for DC differentiation by removing CD3 + , Gr-1 + and B220 + cells using biotinylated anti-Gr-1 (RB6-8C5), biotinylated anti-CD3 (17A2) and biotinylated anti-B220 (RA3-6B2) antibodies (BioLegend) with magnetic streptavidin MACS beads (Miltenyi). The pre-enriched bone marrow cells were cultured with 20 ng/mL GM-CSF (PeproTech) for 7–10 days, and harvested from the flasks and enriched for CD11c + bone marrow-derived dendritic cells (BMDCs) using CD11c MACS beads (Miltenyi) on the final day of culture. BMDCs were stained with a deep red cell tracker (Thermo Fisher). The spleen was harvested from mice (between 7 and 10 weeks of age), and T cells were purified from splenocytes using a naive CD8^+^ T cell isolation kit (Miltenyi), CD8^+^ T cell isolation kit (Miltenyi), or naive CD4^+^ T cell isolation kit (Miltenyi). T cells were stained with 2 µM calcein (Thermo Fisher).

### Experimental setup

Purified and fluorescently labeled BMDCs were cultured on fibronectin-coated 8-well chambered cover glass slides (Labtek) at a concentration of 1.6 × 10^5^ cells per well, with 1 µg/mL LPS overnight. The next day, the BMDCs were washed, and media with the 10 µg/mL of the appropriate peptide (cognate wells), or with media alone (non-cognate wells) was added to the BMDCs. The N4 peptide OVA_257-264_ (SIINFEKL) or variant Q4 peptide OVA_257-264_ (SIIQFEKL) were used for OT-I experiments, the IGRP_206-214_ peptide (VYLKTNVFL) was used for NY8.3 experiments, and the OVA_323-339_ peptide (ISQAVHAAHAEINEAGR) was used for OT-II experiments. Note that the Q4 peptide is reported to have a 18-fold difference in the functional avidity ^27^, and a 6.5 fold difference in the 2D affinity^28^, in comparison to the N4 peptide. After 3 h, certain wells were incubated with 0.01 mg/mL anti-CD40 (HM40-3; BioLegend) antibodies for 10 min. The BMDCs were then washed, and fluorescently labeled, purified CD8^+^ T cells were added to each well at a concentration of 5 × 10^4^ cells per well. These cell densities were selected to ensure contact between T cells and DCs, while enabling accurate tracking of the T cells. The cells were then imaged on a Nikon spinning-disk confocal microscope using a 20 × objective. The cells were maintained in a humid environment with 5% CO_2_ using a Tokai Hit stage-top incubator and objective heater. Time-lapse images were taken of 3 spots per well, every 1–2.5 min for up to 80 min. To assess T cell activation, the protocol was the same, except 1 × 10^5^ BMDCs were co-cultured with 5 × 10^4^ T cells in a 96 well plate for 3 days, at which point the supernatant was collected, and an IL-2 ELISA (R&D Systems) was performed. Note that OT-I and OT-II T cells were cultured with BMDCs harvested from C57BL/6, OT-I or OT-II mice, while NY8.3 T cells were cultured with BMDCs harvested from NY8.3 or NOD mice.

### T cell tracking and video processing

Following image acquisition, the T cells were identified and tracked using the TrackMate plugin on Fiji^29^, and the tracks were used to generate videos in the frame of reference of each individual T cell in MATLAB. A $101 \mathrm{px}\times 101 \mathrm{px}$ region was cropped around the centroid at each timepoint in a track, and concatenated to form a video, with the T cell of interest in the middle. However, for instances where the cells were located at the edge of the original imaged region, the cell is located at the edge of the 101 × 101 pixel frame. All videos had at least 20 frames, and any cells with less than 20 frames were excluded. In order to ensure that only the T cell of interest was visible, any neighboring T cells were removed from each frame by using ROIs of the tracked T cell to apply a mask, which removed everything outside of the ROI, or T cell of interest. The T cells and DC channels were then blurred using the Gaussian blur filter, and then binarized. The DC channel was binarized in Fiji using values determined by the Huang thresholding for the original image, prior to cropping, while the T cell channel was binarized using values determined by Otsu thresholding algorithm, after cropping. A variety of thresholding methods were tested to ensure accurate and complete binarization of all T cells and DCs.

While these 20–61 frame videos were used for the non-deep learning classification methods, classification by deep learning required all videos to have the same number of frames. As such, the videos were evenly sampled to generate videos with 20 frames each. More specifically, in videos with 20–39 frames the first 20 frames were selected, in videos with 40–59 frames, every other frame was selected for a total of 20 frames, and for videos with 60–61 + frames, every third frame was selected for a total of 20 frames. Videos were also generated in which either the first 20, 10, 5, or 2 consecutive frames were selected.

### Non-deep learning quantification

After binarization, Fiji was used to determine the number of red pixels in each frame. The region of overlap between the T cell (green) and DC (red) channel was also visualized and quantified. Based on the time between each frame, TrackMate calculated the T cell speed between each frame along a track, which was used to calculate average speed. Circularity was also calculated in Fiji. Interaction time for each track was calculated by identifying consecutive frames in which red and green pixels were overlapping, representing the T cell interacting with a DC. Due to differences in the total time imaged for each experiment, and for T cells within an experiment, in order to include all of the T cells, interaction time was quantified as the percentage of time each T cell interacts with a DC.

### Deep learning model

The deep learning model is composed of a feature extractor with 4 blocks of time distributed CNN, batch normalization and max pooling layers. The model takes in an input with the shape: (number of frames, 101 (height), 101 (width), 3 (number of channels)). The details of the feature extractor are as follows: 3 × 3 kernel with 8 filters (block 1 conv1), 3 × 3 kernel with 4 filters (block 1 conv2), batch normalization, max pooling with 2 × 2 pool size and 1 × 1 stride, 3 × 3 kernel with 16 filters (block 2 conv1), 3 × 3 kernel with 8 filters (block 2 conv2), batch normalization, max pooling with 2 × 2 pool size and 1 × 1 stride, 3 × 3 kernel with 32 filters (block 3 conv1), 3 × 3 kernel with 16 filters (block 3 conv2), batch normalization, 2 × 2 pool size and 1 × 1 stride, 3 × 3 kernel with 64 filters (block 4 conv1), batch normalization, 2 × 2 pool size and 1 × 1 stride. The extracted features from each frame were flattened and passed to an LSTM layer with 100 units, dropout of 0.2, recurrent dropout of 0.2, and L2 regularizer of 0.01. Finally, the model had a fully connected layer with 100 units, and a softmax classification layer with 2 units. The RMSprop optimizer algorithm, learning rate of 10^–4^, and binary crossentropy loss function were used. The model was trained for 20 epochs using 80% of the videos of cognate and non-cognate OT-I T cells interacting with high affinity N4-DCs or un-pulsed DCs, respectively (with or without aCD40), and validated using 10% of the videos from the same groups. Training data was also randomly augmented via horizontal or vertical shift to increase the size of the dataset. Testing was completed using 10% of the high affinity cognate and non-cognate OT-I T cells, all of the videos of OT-I T cells interacting with Q4-DCs, and all of the videos of NY8.3 T cells.

Finally, to interpret the areas of importance for the designated classification, the Grad-CAMs were visualized as a heat map^21^. This technique uses the gradient of the classification score for a CNN layer (in this case, the first CNN layer in the penultimate CNN block) with respect to the model loss to generate a localization map which indicates the regions of importance for a particular class.

### Statistical analysis

All statistical analysis was performed in Graphpad Prism 8. For comparisons of 2 groups, a Mann–Whitney U test was performed, and for comparisons of more than 2 groups, a Kruskal–Wallis test with Dunn’s test for multiple comparisons was used. Violin plots report the median in the dashed black line, and the quartiles in the dotted colored lines.

## Supplementary Information

Supplementary Information.

## Data Availability

The datasets generated during the current study are available from the corresponding author on reasonable request.
