# Supplementary material for: Rapid video-based deep learning of cognate versus non-cognate T cell-dendritic cell interactions
Source: Sci Rep. 2022 Jan 11;12:559. doi: 10.1038/s41598-021-04286-5 (PMC8752671; doi:10.1038/s41598-021-04286-5)
Supplement: Supplementary file 1 — Supplementary Information. [file 41598_2021_4286_MOESM1_ESM.pdf]

Supplementary information for:

Rapid video-based deep learning of cognate versus non-cognate T cell-dendritic cell interactions

Priya N. Anandakumaran[1], Abigail G. Ayers[1], Pawel Muranski[2,3], Remi J. Creusot[3,4], Samuel K. Sia[1*]

[1] Department of Biomedical Engineering, Columbia University, New York, NY 10027, USA

[2] Department of Medicine, Division of Hematology/Oncology, Columbia University Irving Medical Center, New York, NY, 10032, USA

[3] Columbia Center for Translational Immunology, Columbia University Irving Medical Center, New York, NY, 10032, USA

[4] Department of Medicine and Naomi Berrie Diabetes Center, Columbia University Irving Medical Center, New York, NY, 10032, USA

*Correspondence to be addressed to ss2735@columbia.edu

Supplementary Table S1 – Other methods used to classify antigen-specific T cells

Reference	Experimental setup	Classification method	Cellular feature for classification	Time required to culture T cells	Limited to pre-defined peptide sequences
[1]	Bulk culture	Intracellular cytokine staining	T cell activation	6-24 hours	No
[2]	Bulk culture	Tetramers	TCR specificity	1 hour	Yes
[3]	Trapping of genetically modified cells in microdroplets	Increased fluorescence	T cell activation	9 hours	No
[4]	Flowing cells through microfluidic channel	Velocity differences	TCR-pMHC affinity	Seconds	Yes
[5]	Biopsy samples	Deep learning of morphological variables from static images	Cognate T cell-DC interactions	12 hours of *in vivo* interactions	No
[6]	Bulk culture	Machine learning of autofluorescence lifetime	T cell activation	72 hours	No
Current study	Bulk culture	Deep learning of videos	Cognate T cell-DC interactions	20-80 minutes	*Potentially no

* Unlike multimers or artificial APCs, which require the peptide sequence to be known. In this study, we pulsed DCs with a known peptide sequence, but this approach can potentially be done without this step if the isolated DCs (or APCs) are already presenting antigens (for example, tumor-associated antigens with unknown peptide sequences).

Supplementary Fig S1

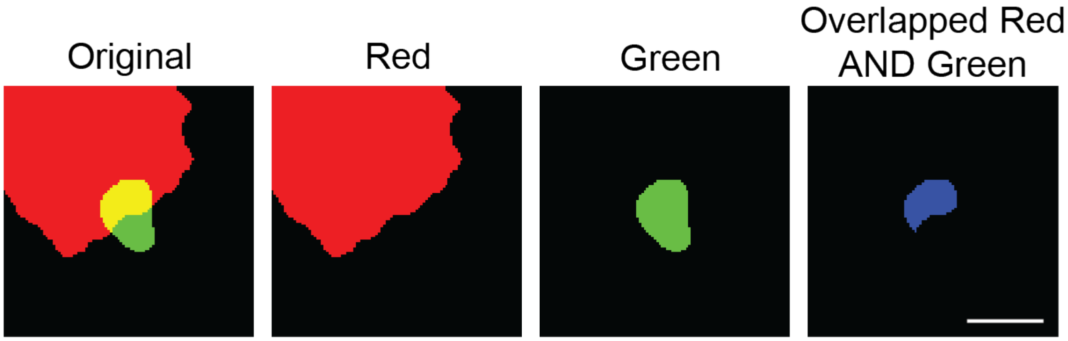

Example frame separated into individual red and green channels, as well as the region of overlap between the red and green channels. These were used to quantify red pixels, and overlapped pixels. Scale bar is 10 µm.

Supplementary Fig S2

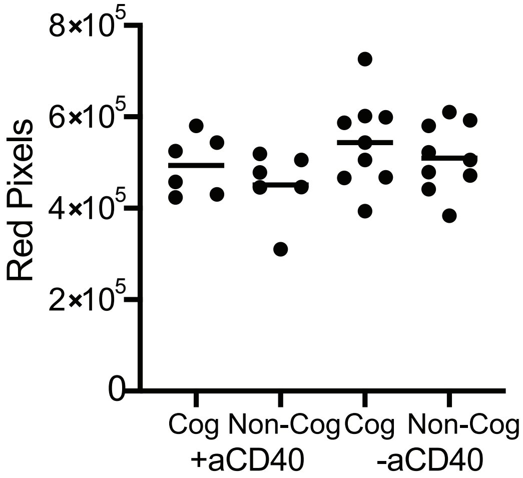

Quantification of red pixels in the original videos. The average number of red pixels over all of the frames in the original videos was calculated in order to determine whether there were differences in the number of red pixels, and thus, the number of DCs, prior to cropping into individual videos.

Supplementary Fig S3

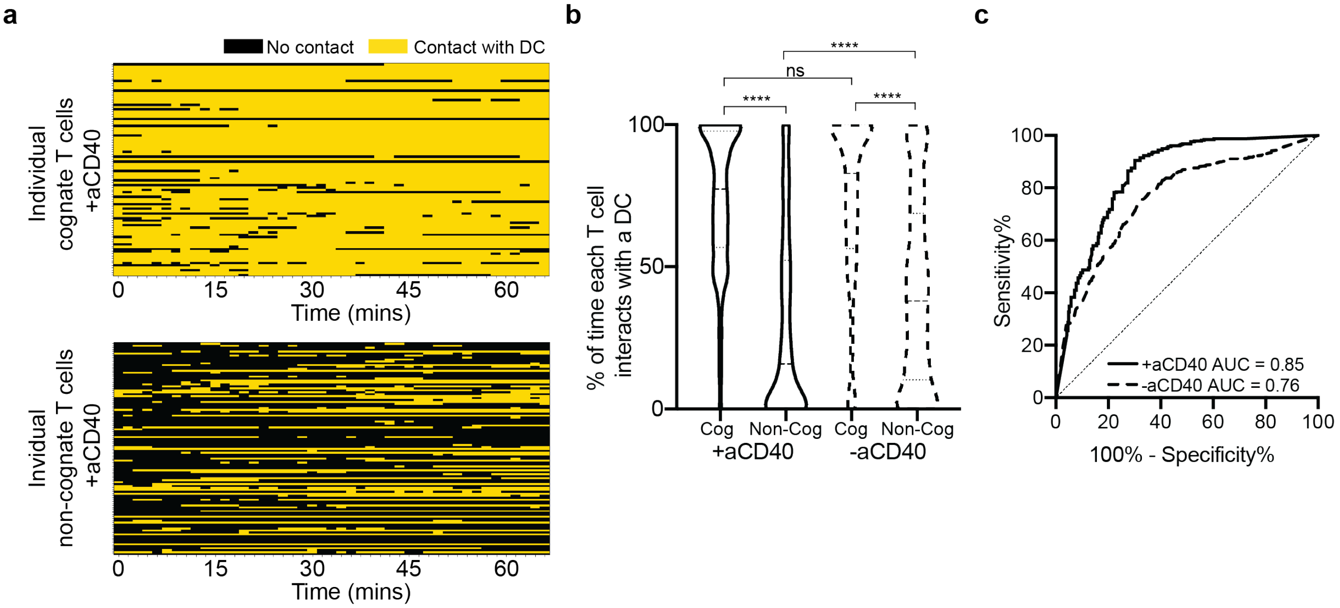

Classifying cognate and non-cognate T cells using a temporal metric. (a) Visual representation of +aCD40 cognate T cell (top) and +aCD40 non-cognate T cell (bottom) interactions with DCs over the course of the experiment. A subset of the T cells which were imaged for around 68 minutes are visualized. This was generated using the overlapped pixel data set, in that a T cell was considered to be interacting with a DC (yellow) if they had some degree of overlap in a frame. (b) Interaction time was quantified as a percentage of time each cognate or non-cognate T cell interacts with DCs, when incubated with or without aCD40. (c) ROC curve quantifying the ability of a temporal metric to discriminate between cognate and non-cognate T cells.

Supplementary Fig S4

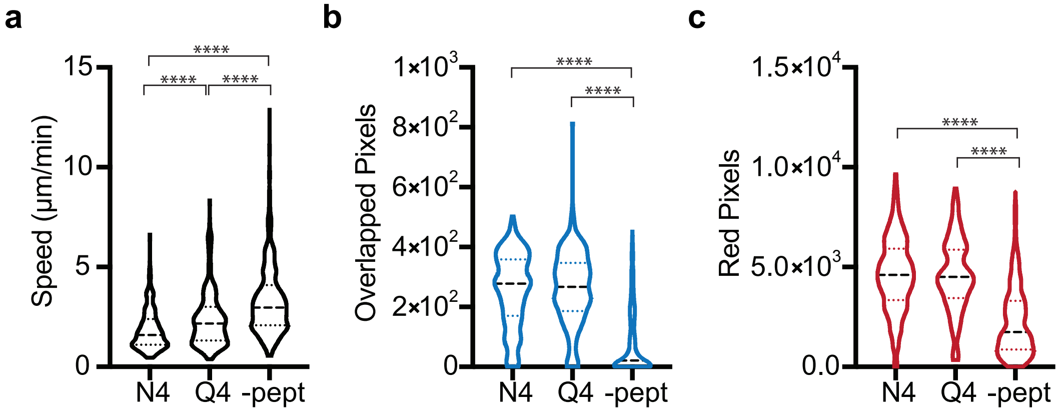

Non-machine learning-based comparison of videos of OT-I cells interacting with high or low affinity cognate DCs, or non-cognate DCs. Data include cognate DCs presenting high affinity N4 peptides (N4), cognate DCs presenting low affinity Q4 peptides (Q4), or non-cognate un-pulsed DCs (-pept) in terms of average (a) T cell speed (b) overlapped pixels and (c) red pixels.

Supplementary Fig S5

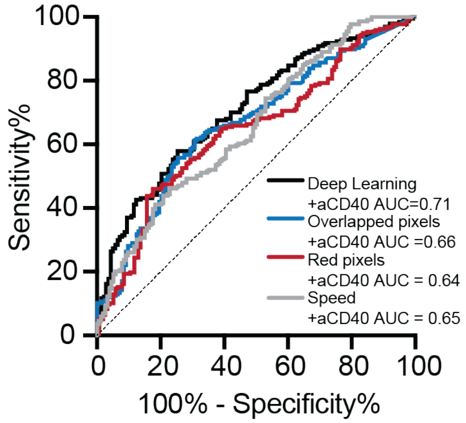

ROC curve quantifying the ability of model, with weights specific to CD8$^+$ T cells from OT-I mice, to classify CD4$^+$ T cell and DC interactions from OT-II mice. +aCD40 deep learning model does not generalize well to CD4$^+$ T cells harvested from OT-II mice. Overlapped pixels, red pixels and speed also provide mediocre discrimination between cognate and non-cognate CD4$^+$ T cell-DCs.

References

1 Linnemann, C. *et al.* High-throughput epitope discovery reveals frequent recognition of neo-antigens by CD4+ T cells in human melanoma. *Nature medicine* **21**, 81-85 (2015).

2 Cohen, C. J. *et al.* Isolation of neoantigen-specific T cells from tumor and peripheral lymphocytes. *The Journal of clinical investigation* **125**, 3981-3991 (2015).

3 Segaliny, A. I. *et al.* Functional TCR T cell screening using single-cell droplet microfluidics. *Lab on a Chip* **18**, 3733-3749 (2018).

4 Stockslager, M. A. *et al.* Microfluidic platform for characterizing TCR–pMHC interactions. *Biomicrofluidics* **11**, 064103 (2017).

5 Liarski, V. M. *et al.* Quantifying in situ adaptive immune cell cognate interactions in humans. *Nature immunology* **20**, 503-513 (2019).

6 Walsh, A. J. *et al.* Classification of T-cell activation via autofluorescence lifetime imaging. *Nature biomedical engineering* **5**, 77-88 (2021).
